# Exploring the potential role of PD-L2 in primary melanoma immunotherapy resistance: a hypothesis-driven dual-ligand framework

**DOI:** 10.3389/fimmu.2026.1942224

**Published:** 2026-10-19

**Authors:** Wenjing Song, Yangyang Tao, Xiang Ji, Wanting Fu, Xin Cai, Xiu Shan

**Affiliations:** Dalian Medical University, The First Affiliated Hospital, Department of Oncology, Dalian, Liaoning, China

**Keywords:** immune checkpoint blockade, melanoma, predictive biomarker, primary immune resistance, programmed death-1, programmed death-ligand 2, repulsive guidance molecule b

## Abstract

**Background:**

The treatment of melanoma has been significantly improved by immune checkpoint blockade (ICB); however, primary immune resistance, particularly in PD-L1-negative tumors and in selected treatment contexts, remains a critical efficacy bottleneck. Traditional biomarkers show clear limitations in explaining resistance mechanisms and guiding clinical stratification.

**Objective:**

This review systematically proposes and elucidates the dual-ligand resistance model, comprehensively examines the molecular structure of programmed death-ligand 2 (PD-L2), its distinct dual-receptor interaction pattern with programmed death-1 (PD-1) and repulsive guidance molecule b (RGMb), and its multi-layered regulatory network, and explores the potential clinical value of PD-L2 as a context-dependent biomarker for primary immune resistance in melanoma and a potential therapeutic target.

**Results:**

Within this framework, PD-L2 may contribute to immune escape in selected contexts, particularly in PD-L1-negative tumors or after PD-L1-targeted therapy. The expression of PD-L2 is regulated by genomic, epigenetic, microenvironmental, and host factors, with evidence of subtype-specific patterns. Available data suggest that PD-L2 may provide complementary biomarker value, although supporting evidence remains heterogeneous and prospective validation is limited. Targeting the PD-L2/RGMb axis has shown potential to reverse primary resistance in preclinical models, supporting further investigation.

**Conclusion:**

This synthesis positions PD-L2 as a candidate compensatory checkpoint in primary immune resistance in melanoma within a proposed dual-ligand resistance framework. However, its role should be interpreted as context dependent rather than universal. Further melanoma-specific and clinically validated studies are needed to define its biomarker and therapeutic value.

## 1 Introduction

The advent of immune checkpoint inhibitors (ICIs) has revolutionized melanoma treatment. By targeting cytotoxic T-lymphocyte-associated protein 4 (CTLA-4) and the programmed cell death protein 1 (PD-1)/programmed death-ligand 1 (PD-L1) axis, ICIs restore T cell anti-tumor immunity, significantly improving outcomes and enabling long-term survival in advanced melanoma (1). Early CTLA-4 blockade studies confirmed that pre-existing immune activity in the tumor microenvironment is key to patient benefit (2). Importantly, PD-L1-negative tumors, anti-PD-L1 therapy, and anti-PD-1 therapy represent distinct biological and therapeutic contexts.

However, primary immune resistance, defined as lack of initial response, remains a major challenge. Even with first-line anti-PD-1 therapy, 30%–35% of patients with advanced melanoma show primary resistance, rising to 50%–80% in refractory subtypes such as acral, mucosal, and uveal melanoma (3). Benefit rates are severely limited in these subtypes (4). Conventional strategies targeting the PD-1/PD-L1 axis provide limited benefit in PD-L1-negative tumors, and this unmet need also extends across later-line settings such as tumor-infiltrating lymphocyte (TIL) therapy, where primary resistance remains common in PD-L1-negative patients from the outset of treatment (5, 6). This complexity of resistance stems from the intertwined nature of immune checkpoint pathways and intrinsic tumor cell signaling networks (7). Traditional biomarkers such as PD-L1 expression and tumor mutational burden (TMB) have clear limitations in explaining resistance mechanisms and guiding clinical practice (8, 9). Moreover, some PD-L1-negative patients still derive benefit from ICIs, whereas certain PD-L1-high patients exhibit resistance. These observations suggest that loss or blockade of PD-L1 function may, in selected contexts, be accompanied by compensatory immunosuppressive mechanisms in the tumor microenvironment. The core mechanism that maintains immune suppression in PD-L1-negative tumors therefore remains incompletely defined. PD-L1 negativity should not be equated with an immune-desert phenotype or attributed to a single mechanism. Antigen-presentation defects, abnormalities in IFN-γ signaling, T-cell exclusion, immunosuppressive myeloid populations, TGF-β and WNT/β-catenin signaling, metabolic constraints, and tumor heterogeneity may each contribute to primary resistance. PD-L2 should therefore be regarded as a context-dependent candidate contributor rather than a necessary explanation for immune suppression in PD-L1-negative melanoma.

Programmed death-ligand 2 (PD-L2) offers critical insights. Unlike PD-L1, PD-L2 has a 2- to 6-fold higher affinity for PD-1 and uniquely possesses a second functional receptor, repulsive guidance molecule b (RGMb) (10). Structural studies show that the binding sites for PD-1 and RGMb are spatially independent, which has led to the hypothesis that PD-L2 may sustain immunosuppressive signaling through PD-1- and RGMb-associated pathways in tumors with low or absent PD-L1 expression, or following PD-L1 blockade, potentially contributing to T-cell dysfunction (11). Multiple lines of evidence support PD-L2 as a potential complementary biomarker for ICB response: PD-L1-negative/PD-L2-positive patients derive significant benefit (12); anti-PD-L2 treatment reverses resistance in preclinical models (13); and high PD-L2 expression correlates with cluster of differentiation 8-positive T cell (CD8^+^ T cell) infiltration and interferon-gamma (IFN-γ) signaling, suggesting that PD-L2 may reflect an inflamed or adaptive immune-resistance phenotype rather than directly indicating treatment resistance (14).

Nevertheless, current studies largely view PD-L2 as an auxiliary molecule to PD-L1, and a systematic framework remains lacking to define the contexts in which PD-L2 may contribute to immune suppression in selected PD-L1-negative tumors, alongside alternative mechanisms of resistance. This review synthesizes available evidence into a dual-ligand resistance model as a working theoretical framework for primary immune resistance in PD-L1-negative melanoma. Rather than replacing the conventional PD-1/PD-L1 single-axis paradigm, this model integrates PD-L2 and its dual-receptor mechanism as a potential compensatory layer within the broader landscape of immune resistance. Based on this model, we address four key questions: How do PD-L2’s molecular structure and dual-receptor interactions confer its unique compensatory function? Through which multi-layered regulatory networks is PD-L2 expression shaped? How do PD-L2 regulatory patterns differ across melanoma subtypes and explain differential resistance? And how can PD-L2 be translated into clinically useful predictive biomarkers and therapeutic targets? We anticipate that this model will deepen understanding of melanoma immune evasion and subtype-specific resistance, while providing a mechanistic and translational framework to guide future validation in PD-L1-negative refractory melanoma.

## 2 Dual-ligand resistance model

Based on structural and functional differences between PD-L1 and PD-L2 in melanoma immune resistance, we propose that these ligands may contribute differently to immune regulation. Their distinct molecular architectures and receptor-binding patterns provide a rationale for the hypothesis that PD-L2 may act as a compensatory pathway in selected contexts where PD-L1 function is reduced or blocked. Building on this rationale, this review formally proposes the dual-ligand resistance model as a working framework to explain primary immune resistance in PD-L1-negative melanoma. This framework is supported by preclinical evidence from independent studies (11, 13), but the relative contribution of the PD-1 and RGMb axes, and how this balance may vary across melanoma subtypes, remains to be defined. In this chapter, we first compare the molecular structure, receptor interactions, and signal transduction features of PD-L1 and PD-L2, then outline the basis of the dual-ligand resistance model, and finally discuss how PD-L2 may compensate in contexts where PD-L1 signaling is diminished. The structural features of PD-L2 that determine its dual-receptor binding capacity and immunosuppressive function are depicted in **Figure 1**.

**Figure 1:**
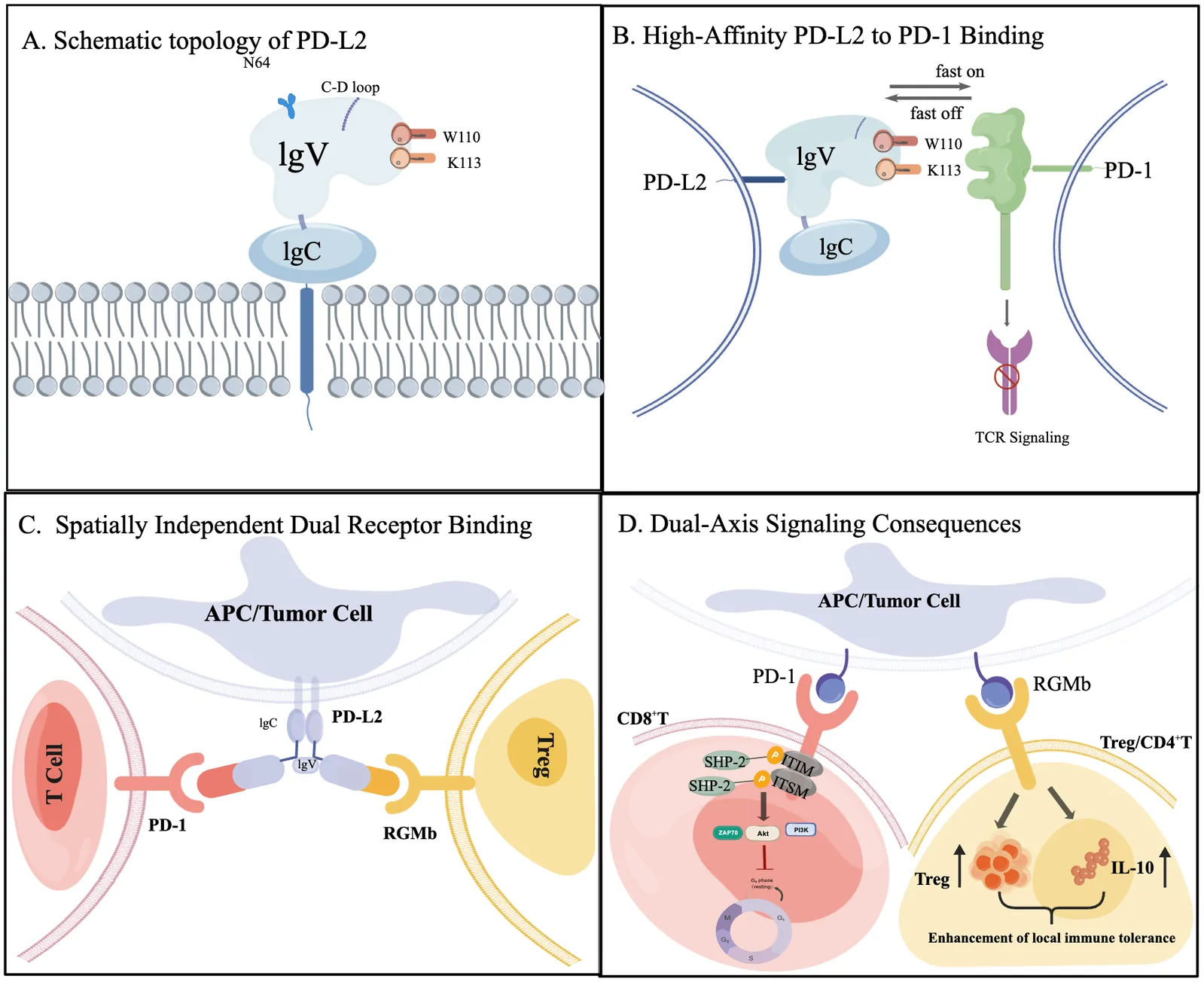
Molecular structure, dual receptor binding and signal transduction of PD-L2. **(A)** Schematic topology of PD-L2, showing its extracellular IgV and IgC domains, transmembrane region, intracellular tail, and PD-L2 residues W110 and K113 located within the PD-1-binding interface. **(B)** High-affinity binding of PD-L2 to PD-1, illustrating the interaction between PD-L2 and PD-1 and the downstream inhibitory signaling mediated through PD-1. **(C)** Spatially independent dual-receptor binding of PD-L2, showing that PD-L2 can interact with PD-1 on T cells and RGMb on regulatory T cells through distinct binding interfaces. **(D)** Dual-axis signaling consequences of PD-L2 engagement, showing PD-1-mediated inhibition of effector T-cell activity and RGMb-associated immune regulatory signaling in regulatory T cells, including enhanced IL-10 production and local immune tolerance. Created with BioGDP.com.

### 2.1 The structural characteristics of PD-L1 and the binding characteristics of PD-1

PD-L1 (CD274/B7-H1), the primary ligand of PD-1, is a type I transmembrane protein encoded by the CD274 gene, with an extracellular region containing an IgV and an IgC domain (15). Crystal structures of the PD-1/PD-L1 complex reveal that their IgV domains pair in a manner analogous to antibody Fv regions, forming a 1:1 receptor-ligand complex (16). The core function of PD-L1 is to initiate inhibitory signaling in T cells by binding to the IgV domain of PD-1. This interaction induces phosphorylation of the immunoreceptor tyrosine-based inhibition motif (ITIM) and immunoreceptor tyrosine-based switch motif (ITSM) in the cytoplasmic tail of PD-1, leading to recruitment of Src homology region 2 domain-containing phosphatases 1 and 2 (SHP-1/2). Through dephosphorylation, these phosphatases block key downstream signaling molecules of the T cell receptor (TCR) pathway, including zeta-chain-associated protein kinase 70 kDa (ZAP70) and phosphoinositide 3-kinase/protein kinase B (PI3K/Akt), ultimately suppressing T cell proliferation, cytokine secretion, and effector function activation (17, 18).

PD-L1 exhibits slow association and slow dissociation kinetics toward PD-1, coupled with notable conformational changes, a feature not shared by PD-L2 (19). PD-L1 also uniquely binds B7-1 (CD80), with a dissociation constant of approximately 1.7 μM (20). This interaction partially overlaps with the PD-1/PD-L1 binding interface and may modulate inhibitory signaling within the tumor microenvironment. In contexts where PD-L1 signaling is reduced or blocked, PD-L2 may become more relevant as a compensatory checkpoint, which provides a structural basis for the dual-ligand resistance model.

### 2.2 Fine molecular structure and dual receptor interaction mechanism of PD-L2

PD-L2 (CD273/B7-DC), the second natural ligand of PD-1, is a type I transmembrane glycoprotein that uses its extracellular IgV domain for receptor recognition (10, 21).PD-L2 expression is regulated by nuclear factor kappa-light-chain-enhancer of activated B cells (NF-κB) and interleukin-4/signal transducer and activator of transcription 6 (IL-4/STAT6), and its physiological expression has been described in medullary thymic dendritic cells (22). The IgV domain of PD-L2 differs from PD-L1 in sequence composition, loop architecture, and key contact sites. These structural characteristics determine its distinct binding kinetics and dual-receptor mode of action.

#### 2.2.1 High-affinity binding to PD-1 and its structural basis

PD-L2 binds PD-1 with a dissociation constant (Kd) of 26–37 nM, which is 2- to 6-fold higher than that of PD-L1. This difference is mainly attributed to its slower dissociation rate (koff = 0.0018–0.0032 s^–1^) (10).

The structure of the murine PD-1/PD-L2 complex (PDB 3BP5) reveals that the two molecules form a stable 1:1 interface via immunoglobulin variable (IgV) domains, with a buried surface area of 1950 Å^2^. Residues W110 and K113 of PD-L2 are critical for high-affinity interaction and constitute the core binding region (23).

The high-resolution human PD-1/PD-L2 structure (2.0 Å) further demonstrates that PD-1 forms an induced deep pocket of 170 Å^3^ upon binding to PD-L2, larger than the 80 Å^3^ shallow cavity formed during PD-L1 engagement. This structural feature provides a basis for drug design. The CC′ loop of PD-1 intrinsically adopts a ligand-binding conformation, enabling PD-L2 to rapidly bind and stabilize the active state of PD-1. These structural features explain why PD-L2 may be capable of sustaining PD-1 signaling under conditions where PD-L1 is diminished or blocked (23, 24).

Distinct from PD-L1, PD-L2 interacts with PD-1 via fast association and fast dissociation without substantial conformational rearrangement. In this context, PD-L2 may occupy PD-1 when PD-L1 signaling is reduced, but this should be interpreted as a mechanistic hypothesis rather than direct evidence of melanoma resistance. In addition, the N64 glycosylation site of PD-L2 modulates receptor binding affinity and may influence pathway activity (10, 19, 25).

#### 2.2.2 Unique dual-receptor mechanism of RGMb and spatial independence

PD-L2 is the only known immune checkpoint ligand capable of binding both PD-1 and RGMb, a function absent in PD-L1. This dual-receptor mode arises from the structural plasticity of its IgV domain (21, 26).

Its binding sites for PD-1 and RGMb are spatially independent and non-competitive. PD-L2 can engage the two inhibitory pathways separately or simultaneously. In melanoma, this raises the possibility that PD-L2 may preserve immunoregulatory signaling through RGMb during anti-PD-1 therapy; however, the dominant contribution of this axis in human melanoma remains to be established. The IgV domain alone mediates specific binding to RGMb, which does not interact with PD-L1 (11, 26, 27).

In addition, the PD-L2-binding interface on RGMb is separate from that for bone morphogenetic protein 2/4 (BMP-2/4), avoiding signaling crosstalk and supporting pathway specificity (26). The functional relevance of this structural independence has been shown in preclinical melanoma models, where selective disruption of PD-L2/RGMb interactions restored anti-tumor responses to anti-PD-L1 therapy, whereas blocking PD-1/PD-L2 alone was insufficient; however, these findings should be viewed as preclinical support rather than proof of a dominant melanoma resistance mechanism (11).

### 2.3 Functional consequences of PD-L1 and PD-L2 signaling

Both PD-L1 and PD-L2 bind PD-1 to mediate immune suppression, yet structural and functional studies suggest differences in binding kinetics and signaling behavior. Ligand-induced conformational changes differ between PD-L1 and PD-L2 (19, 24). PD-L1 ligation triggers mild and local conformational adjustments of PD-1, whereas PD-L2 binding induces a more substantial conformational rearrangement and forms a deep hydrophobic pocket of approximately 170 Å^3^ (24). These structural differences may contribute to distinct signaling properties, providing a structural basis for their divergent functional effects in immune regulation.

#### 2.3.1 PD-L1 signaling: potent and irreversible T cell exhaustion

The binding of PD-L1 to PD-1 induces phosphorylation of the immunoreceptor tyrosine-based inhibitory motif (ITIM) and immunoreceptor tyrosine-based switch motif (ITSM) in the cytoplasmic tail of PD-1, thereby recruiting Src homology region 2 domain-containing phosphatases 1 and 2 (SHP-1/2). This process inhibits key downstream T cell receptor (TCR) signaling molecules, including ZAP70 and PI3K/Akt, and suppresses activation of NFAT and mTOR pathways. As a result, T-cell proliferation, cytokine secretion, and effector function are reduced (17, 18). In tumor settings, PD-L1 contributes to sustained immune suppression, although the degree to which this state is reversible depends on cellular differentiation status and microenvironmental context.

#### 2.3.2 PD-L2 signaling: rapid and reversible dual-axis immune suppression

Unlike PD-L1, which recruits SHP-1/2 phosphatases upon PD-1 engagement, PD-L2 recruits SHP-2 phosphatases (28). Further imaging studies have shown that PD-L2 engagement can induce the formation of an inhibitory signalosome and, through this complex, promote dephosphorylation of the TCR/CD3 complex and downstream signaling molecules (74). In non-melanoma systems, PD-L2–PD-1 engagement has been reported to inhibit TCR-mediated proliferation and cytokine production in CD4^+^ T cells and to arrest cells in G0/G1 without increasing cell death (28). Together with the established concept that PD-1 pathway blockade can reverse CD8^+^ T-cell exhaustion (75), these observations have led to the hypothesis that PD-L2-mediated suppression may be functionally distinct from PD-L1-mediated suppression and more readily reversible in some settings. However, whether this reversibility applies within the melanoma microenvironment—where inflammatory stimuli such as TNF-α can drive reversible phenotypic changes in melanoma cells (76) and where PD-L2 is inducible on tumor cells—remains to be directly investigated. Moreover, the extent to which T-cell function can be restored under inflammatory conditions within tumors has yet to be determined. Pending such validation, the proposed role of PD-L2 as a “flexible” compensatory checkpoint, while mechanistically plausible, should be considered a hypothesis requiring experimental verification in melanoma.

Notably, PD-L2 also establishes dual-axis immune suppression via its unique dual-receptor pattern. In the PD-1-dependent axis, PD-L2 restricts the activation and effector responses of CD8^+^ T cells (28). In the RGMb-dependent axis, PD-L2 independently enhances interleukin-10 (IL-10) production by CD4^+^ T cells and regulatory T cells (Tregs) and promotes Treg expansion, further consolidating local immune suppression (11, 26, 27, 29). In melanoma, these findings suggest a potential compensatory pathway, but the relative contribution of the PD-1 and RGMb axes in human disease remains to be defined. Accordingly, PD-L2 may contribute to immune resistance in PD-L1-low or PD-L1-negative contexts, but current evidence does not establish it as a dominant melanoma resistance mechanism.

#### 2.3.3 Clinical implications of differential signaling patterns

Taken together, these signaling features suggest that PD-L2 may be relevant in selected PD-L1-low, PD-L1-negative, or PD-L1 blockade. However, the clinical evidence in melanoma remains heterogeneous, and most supporting data derive from non-melanoma or preclinical models. Associations between high PD-L2 expression and treatment resistance should therefore be interpreted cautiously, because elevated PD-L2 may also reflect an inflamed or adaptive immune-resistance phenotype rather than direct evidence of causation. In this context, **Figure 2** summarizes the conceptual relationship between PD-L1 and PD-L2 signaling. Likewise, mutations in PDCD1 may alter responsiveness to ICB, but their interaction with PD-L2 in melanoma requires further validation (30).

**Figure 2:**
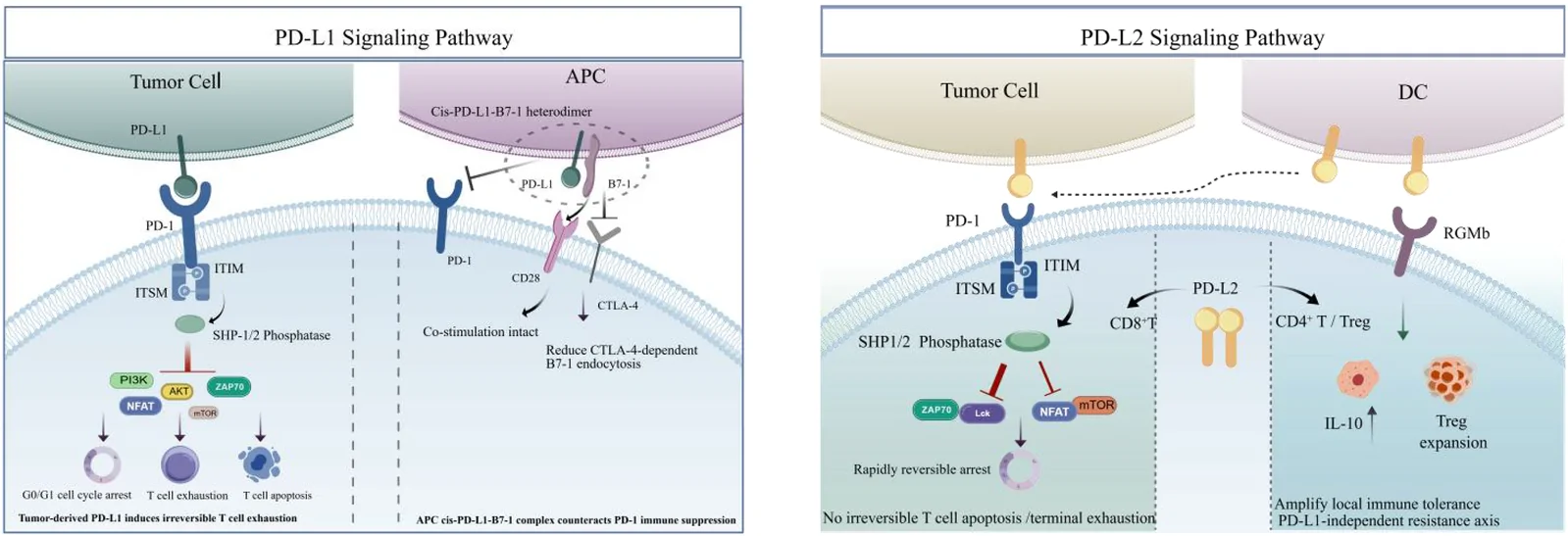
Comparison of PD-L1 and PD-L2 signaling pathways. Created with BioGDP.com.

In summary, PD-L1 and PD-L2 exhibit fundamental differences in molecular structure, PD-1 binding-induced conformational changes, receptor interaction modes, signaling properties, and effects on T-cell function. These differences suggest that the two ligands may contribute differently to immune regulation and provide a structural and functional basis for the dual-ligand resistance model in melanoma. The key features and functional differences between PD-L1 and PD-L2 are systematically summarized in **Table 1**.

**Table 1:** Comparison of PD-L1 and PD-L2 in the proposed dual-ligand resistance framework.

Characteristic dimension	PD‑L1 (CD274, B7‑H1)	PD‑L2 (CD273, B7‑DC)	Proposed role in dual‑ligand resistance framework
Binding kinetics	Slow association and slow dissociation	Higher affinity for PD-1; relatively rapid association and dissociation	Potential compensatory occupancy; may facilitate PD-1 engagement under selected conditions
Unique receptor	Binds B7-1/CD80	Binds RGMb	May contribute to continued immune regulation through an additional receptor-associated pathway
Signal reversibility	Sustained inhibitory effects; reversibility is context dependent	Potentially reversible functional suppression in some experimental settings	Functional consequences may vary according to ligand and cellular context
T cell effects	Inhibits T-cell activation and effector function through PD-1	May inhibit T-cell responses through PD-1 and regulate CD4^+^ T-cell/Treg-associated functions through RGMb in selected models	Potentially complementary effects on effector T cells and regulatory immune populations
Clinical compensatory relevance	Established therapeutic target in melanoma; expression may be constitutive or inflammation-induced	Candidate biomarker and therapeutic target under investigation; relevance should be assessed separately in PD-L1-low or PD-L1-negative tumors and after PD-L1 blockade	Candidate resistance-related pathway requiring context-specific melanoma validation

## 3 PD-L2 regulatory network: from molecular mechanisms to melanoma subtype specificity

The previous chapter outlined the dual-receptor features of PD-L2 and the structural basis of the dual-ligand resistance model. In melanoma, PD-L2 is regulated by an integrated network involving genomic and epigenetic alterations, microenvironmental inflammatory cues, and host-related factors such as microbiota and senescence (**Figure 3**).

**Figure 3:**
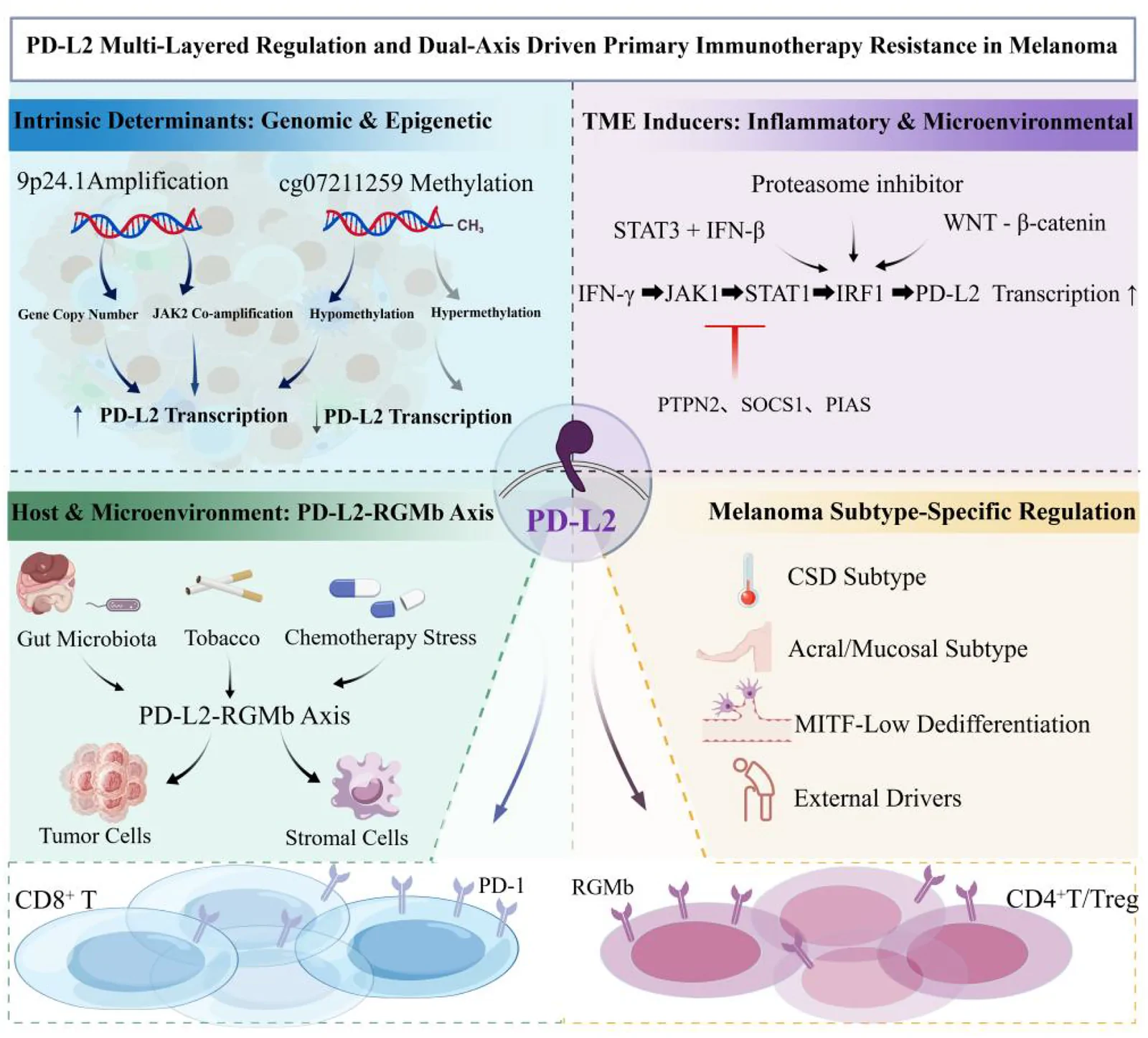
PD-L2 multi-layered regulation and dual-axis driven primary immunotherapy resistance in melanoma. Created with BioGDP.com.

This chapter characterizes upstream regulators of PD-L2, its cellular sources, and subtype-dependent expression patterns, and examines how these factors may shape PD-L2-associated immune resistance. We organize the regulatory system into four modules to clarify the main sources of PD-L2 upregulation. Together, these mechanisms define the expression landscape of PD-L2 and may influence the conditions under which the dual-ligand resistance model becomes relevant. In selected contexts, including PD-L1-negative tumors or PD-L1 blockade, events such as 9p24.1 amplification, cg07211259 hypomethylation, and persistent IFN-γ signaling may contribute to PD-L2 upregulation and immune escape in melanoma.

### 3.1 Genomic regulation: 9p24.1 alterations as a rare molecular context

The 9p24.1 locus contains several immune-regulatory genes, including *CD274* (PD-L1), *PDCD1LG2* (PD-L2), and *JAK2*. Copy-number alterations involving this region have been described in lymphoid malignancies and in a small subset of solid tumors, where they may influence PD-1 ligand expression through increased gene dosage and associated signaling pathways (31). However, most of the established evidence concerns *CD274*/PD-L1 amplification or non-melanoma malignancies. In melanoma, Gupta et al. reported two metastatic cases, one cutaneous and one mucosal, harboring *PD-L1/PD-L2/JAK2* amplification at 9p24.1; both patients experienced durable responses to immunotherapy (32). Given the very small number of cases, this observation does not establish a causal role for PD-L2 in treatment resistance or demonstrate that 9p24.1 amplification is a predictive biomarker.

More broadly, chromatin organization and enhancer-associated regulation may influence the transcription and cell-surface presentation of PD-1 ligands in cancer cells (33). Nevertheless, direct melanoma-specific evidence linking 9p24.1 amplification or rearrangement to PD-L2 overexpression and primary immunotherapy resistance remains limited. Accordingly, 9p24.1 alterations should be regarded as a rare molecular context that warrants further investigation rather than as a general mechanism of melanoma resistance. Genome-wide copy-number loss at high burden has also been associated with downregulation of immune-related genes and resistance to immune checkpoint blockade, although this observation is not specific to PD-L2 (34).

### 3.2 Epigenetic regulation: a core switch mediated by promoter methylation

DNA methylation at promoter regions serves as a central epigenetic mechanism for transcriptional silencing, and hypermethylation of CpG islands around transcription start sites generally induces stable gene repression (35). Melanoma progression is accompanied by widespread epigenetic reprogramming of DNA methylation (36). The methylation level of the cg07211259 locus is associated with PD-L2 transcription (37). Hypomethylation (≤9.92%) at this site is associated with robust PD-L2 mRNA and protein expression, whereas hypermethylation is associated with transcriptional silencing, in part through recruitment of repressive factors such as histone deacetylase (HDAC) (37).

Analysis of 470 melanoma cases from the TCGA cohort reveals a strong negative correlation between cg07211259 methylation and PD-L2 mRNA expression (Spearman’s ρ = -0.43, P < 0.001). Evaluation of 37 melanoma cell lines further suggests that intrinsic PD-L2 expression is linked to promoter methylation status (37). tumor cells with highly methylated PD-L2 promoters fail to significantly upregulate PD-L2 even upon exogenous interferon-γ (IFN-γ) stimulation, suggesting that epigenetic priming contributes to PD-L2 transcriptional inducibility (38).

Genomic amplification and epigenetic modification help define the basal expression landscape and expression potential of PD-L2. On this basis, dynamic signaling cues within the tumor microenvironment may further fine-tune PD-L2 expression in response to external stimuli. This promoter methylation-dependent regulatory mechanism may influence the conditions under which PD-L2-mediated compensatory immune suppression becomes relevant in melanoma.

### 3.3 Microenvironment-induced regulation

Inflammatory signals within the tumor microenvironment regulate PD-L2 expression through multiple interconnected mechanisms, including epigenetic licensing, transcriptional activation, negative feedback, and alternative bypass pathways.

#### 3.3.1 Epigenetic licensing and the canonical transcriptional induction axis

Inflammatory induction of PD-L2 transcription appears to depend on epigenetic permissiveness. Promoter hypermethylation may impose epigenetic silencing of PD-L2 inducibility, whereas promoter demethylation may relieve this constraint and permit IRF1-mediated transactivation. Hypermethylated melanoma cell lines (BNSKCM-01/03) failed to upregulate PD-L2 even upon potent IFN-γ stimulation, suggesting that chromatin accessibility contributes to inflammatory responsiveness at the PD-L2 locus (37). Deletion of the IRF1-binding site abrogated IFN-γ-inducible PD-L2 expression, indicating that IRF1 occupancy is required for transcriptional activation (38). Together, these findings suggest that epigenetic state and inflammatory signaling jointly shape PD-L2 inducibility in contexts where the tumor microenvironment is permissive.

Building on this epigenetic permissiveness, IFN-γ drives PD-L2 transcription through the JAK1–STAT1–IRF1 axis (37–40). In melanoma models, JAK1 has a dominant role in IFN-γ signaling, whereas JAK2 may be dispensable in some experimental contexts (39). Negative-feedback mechanisms may constrain JAK-STAT signaling, but their specific effects on PD-L2 regulation in melanoma remain to be established. Direct evidence concerning PTPN2 as an immunotherapy modulator in melanoma is discussed separately (79, 80). In addition, a JAK-independent activation mode has been described in cancer models: proteasome inhibitors can block IRF1 ubiquitin-mediated degradation, promoting IRF1 nuclear accumulation and sustained PD-L2 transcription (41). Whether this mechanism operates in melanoma and in individual melanoma subtypes requires further validation.

#### 3.3.2 Extended transcriptional regulation and stromal cell regulatory networks

The JAK1–STAT1–IRF1 axis described above represents a major pathway for inflammatory PD-L2 responses; however, PD-L2 transcription is not confined to this single route, and additional regulatory mechanisms may also contribute to its fine-tuned control.

Transcriptional regulation of PD-L2 does not rely exclusively on IRF1. Garcia-Diaz et al. demonstrated, through promoter mutagenesis and ChIP assays, that STAT3 can directly bind the PD-L2 promoter, and this binding was particularly prominent upon IFN-β stimulation; in contrast, no STAT3 binding was detected on the PD-L1 promoter (42). These findings suggest a dual transcription factor regulatory mode for PD-L2, enabling responsiveness to both IFN-γ via IRF1 and IFN-β via STAT3. By comparison, PD-L1 appears to rely predominantly on IRF1 and responds more weakly to IFN-β.

Ravindran Menon et al. (43) found that EGCG downregulates IFN-γ-induced PD-L1 and PD-L2 expression simultaneously in melanoma cells by suppressing the JAK–STAT1–IRF1 axis. Guo et al. (44) systematically reviewed transcriptional regulation of PD-L1 and PD-L2 by the IFN-γ–JAK–STAT pathway in melanoma, emphasizing that pathway integrity is an important determinant of response to immune checkpoint inhibitors. Furthermore, Laurin et al. reported that low expression of PGC1β in melanoma upregulates PD-L2 through activation of the MEK–IRF1 axis, suggesting that mitochondrial dysfunction may also participate in PD-L2 transcriptional regulation (45).

Regulation of PD-L2 by IFN-γ in the tumor microenvironment may be bidirectional. On one hand, IFN-γ induces PD-L2 expression on tumor cells via the JAK1–STAT1–IRF1 pathway, consistent with a negative feedback loop of immune surveillance (37, 39). On the other hand, studies in non-small cell lung cancer have shown that this pathway can also activate stromal cells. IFN-γ drives cancer-associated fibroblasts toward an antigen-presenting phenotype (apCAFs), making them a non-tumor source of PD-L2 (29). Together, these findings suggest that IFN-γ-mediated regulation of PD-L2 may involve both tumor and stromal compartments.

#### 3.3.3 WNT/β-catenin pathway activation and dendritic cell deficiency: a microenvironmental context for PD-L2 regulation

The preceding sections have described direct transcriptional regulation of PD-L2 by inflammatory signals. In addition, compensatory upregulation of PD-L2 may also be associated with a “cold” tumor microenvironment shaped by aberrant activation of the WNT/β-catenin pathway.

Intrinsic activation of the WNT/β-catenin signaling pathway in tumor cells is a recognized driver of the cold tumor phenotype in melanoma and other solid malignancies. Upon pathway activation, nuclear translocation of β-catenin represses CCL4 transcription, impairing recruitment of Batf3^+^ CD103^+^ dendritic cells (DCs) and limiting cross-priming of CD8^+^ T cells, thereby contributing to a T-cell-excluded tumor microenvironment associated with primary resistance to immune checkpoint inhibitor therapy (46, 47). Related associations between activated WNT/β-catenin signaling and reduced immune infiltration have also been reported in human colorectal cancer and advanced thyroid cancer (48, 49), while pan-cancer analyses have linked WNT/β-catenin hyperactivation to a non-T-cell-inflamed phenotype across multiple tumor types (50).

In WNT/β-catenin-driven cold tumors, the paucity of CD103^+^ DCs may impair T-cell priming. WNT/β-catenin pathway activation has also been associated with elevated PD-L2 expression in melanoma specimens. Pinczewski et al. reported that PD-L1 and PD-L2 expression by tumor cells was significantly higher in melanomas with high pS33-β-catenin expression (77), suggesting an association between active WNT/β-catenin signaling and PD-L2 upregulation in human melanoma. Residual antigen-presenting cells may respond to inflammatory signals and potentially upregulate PD-L2 through IRF1- or STAT3-associated pathways (42). Whether this mechanism operates in DC-deficient melanoma remains unknown. PD-L2 derived from multiple cellular sources may therefore contribute to local immune suppression. However, whether DC deficiency directly triggers PD-L2 upregulation in melanoma remains to be experimentally defined, and direct evidence for this causal chain is still lacking. Given that tumor cell-intrinsic PD-L2 expression is low in cold tumors, DC-derived PD-L2 represents a possible, but currently unconfirmed, source of local immunoregulation in this context.

Compensatory upregulation of PD-L2 may represent one mechanism by which tumors evade anti-PD-L1 therapy. While promoting PD-L1 degradation has been shown to remodel antitumor immunity and enhance CD8^+^ T-cell cytotoxicity (52), anti-PD-L1 monotherapy may also impose selective pressure that favors compensatory PD-L2 expression. Supporting evidence has been reported in multiple solid tumor types. In hepatocellular carcinoma, increased PD-L2 on antigen-presenting cells after anti-PD-L1 treatment has been linked to clinical benefit in some patients who later switched to anti-PD-1 therapy. In ovarian cancer, PD-L2 overexpression has been associated with poor prognosis, and PD-L2 neutralization has been reported to reverse immunotherapy resistance (53, 54). Collectively, these findings support PD-L2 as a potential compensatory pathway after PD-L1 blockade, but they do not establish it as the dominant escape mechanism in melanoma.

Together, a WNT/β-catenin-associated cold tumor microenvironment may be associated with changes in PD-L2 regulation across multiple cellular compartments. Whether PD-L2 functionally contributes to immune suppression in WNT/β-catenin-driven melanoma remains unknown. This conceptual framework highlights a possible relationship between β-catenin-associated microenvironmental remodeling and PD-L2 regulation, which requires direct experimental validation before any role in ICB resistance can be inferred.

### 3.4 Host–microbiota and cellular crosstalk regulate the PD-L2–RGMb axis

The PD-L2–RGMb axis links host-related factors with tumor immune responses, and its regulation appears to depend on both host context and cell type. This extends the scope of the dual-ligand resistance model from tumor -intrinsic regulation to the broader host microenvironment.

#### 3.4.1 Diversity of host factors: from microbiota to environmental exposure

The PD-L2–RGMb axis adds an additional layer beyond the conventional PD-1/PD-L1 inhibitory paradigm. In physiological settings, this axis helps maintain peripheral immune tolerance. Xiao et al. showed that blocking the RGMb–PD-L2 interaction abrogates respiratory tolerance and induces excessive T-cell proliferation and cytokine release, indicating that this axis can negatively regulate immune responses and contribute to tissue homeostasis under normal conditions (26). In melanoma, these observations provide a rationale for considering the PD-L2–RGMb axis as a potential immune-regulatory pathway, but direct evidence for a dominant role in human disease remains limited.

Recent studies suggest that the gut microbiota may influence the tumor microenvironment through the PD-L2–RGMb axis and thereby affect immunotherapy response in melanoma. Because PD-L2 also functions as a co-receptor in this axis (26, 27), it participates in both effector and tolerance-related processes. In preclinical systems, combined blockade of this axis has been reported to reverse resistance (11, 55). A phase I clinical trial of fecal microbiota transplantation combined with anti-PD-1 therapy also supports the idea that microbiota remodeling can improve ICI response, although multiple mechanisms are likely involved, including enhanced T-cell infiltration and broader immunogenic remodeling (56).

Activation of this axis is not restricted to microbiota-related effects. In non-small cell lung cancer, treatment-related factors such as neoadjuvant chemotherapy and IFN-γ released during immunotherapy, as well as environmental exposures such as tobacco, have been shown to activate the axis. Chemotherapy can induce PD-L2 expression in cancer-associated fibroblasts, which recruit FOXP1^+^ regulatory T cells via RGMb, whereas tobacco exposure can activate a PD-L2/RGMb/NF-κB/CCL20 cascade in tumor cells (57). These findings provide context for how host and environmental factors may regulate PD-L2, but they should not be interpreted as direct evidence for melanoma.

#### 3.4.2 Cell type specificity: from tumor cells to stromal cells

Activation of the PD-L2–RGMb axis appears to show marked cell type dependence, with the predominant ligand source varying across cancer types. In melanoma, the gut microbiota has been reported to regulate PD-L2 expression and RGMb membrane localization in tumor cells (11). In non-small cell lung cancer, chemotherapy-induced IFN-γ can reprogram stromal cells toward an antigen-presenting phenotype (apCAFs), which then serve as a non-tumor source of PD-L2 and mediate immune evasion via RGMb (29). Thus, stromal cells may contribute to the PD-L2 ligand pool under certain microenvironmental conditions. However, whether apCAF-derived PD-L2 occurs in melanoma remains to be validated. Collectively, these findings suggest that the PD-L2–RGMb axis may integrate microbiota, stromal remodeling, and environmental exposures, but its relative importance in melanoma still requires direct study.

### 3.5 Subtype-specific regulatory mechanisms of PD-L2 in melanoma

PD-L2 expression is regulated by both tumor-intrinsic states and tumor microenvironmental signals, with patterns that may vary across melanoma subtypes (58). Based on their proposed driving mechanisms, PD-L2 regulation can be broadly categorized into four patterns: inflammation-associated regulation in the chronic sun-induced damage (CSD) subtype, epigenetically associated regulation in acral and mucosal subtypes, compensatory upregulation associated with the MITF-dedifferentiated phenotype, and externally influenced regulation related to host aging and chemotherapy stress. These patterns differ in their potential driving signals, cellular sources, and therapeutic implications, suggesting that PD-L2-targeted strategies may need to be evaluated according to melanoma subtype and treatment context.

#### 3.5.1 Chronic sun-induced damage subtype: inflammation-driven adaptive regulation

The CSD subtype is characterized by high CD8^+^ TIL infiltration. PD-L2 expression shows a significant positive correlation with TIL infiltration (P < 0.003) and spatial enrichment at the tumor –host interface (58). PD-L2 positivity in this subtype reaches 62%, with mixed/lentiginous subtypes showing higher positivity than pure desmoplastic subtypes (P = 0.047) (58).

These findings are consistent with adaptive induction of PD-L2 by IFN-γ. Infiltrating TILs may secrete IFN-γ, which can promote PD-L2 upregulation through an intact JAK–STAT pathway, forming a potential “TIL infiltration → IFN-γ → PD-L2” negative-feedback loop (38). Thus, PD-L2 expression in the CSD subtype may represent a dynamic response to inflammatory signals rather than a fixed molecular state.

This regulatory pattern provides a rationale for investigating upstream pathway modulation, including JAK inhibition, as a potential approach to alter inducible PD-L2 expression. However, whether such interventions can improve treatment response or produce clinical benefit in this subtype remains to be determined through prospective studies.

#### 3.5.2 Acral/mucosal subtype: epigenetically preprogrammed sustained suppression

Acral, mucosal, and melanoma of unknown primary (MUP) melanomas commonly exhibit low TIL and IFN-γ levels and limited responsiveness to standard immunotherapies (58, 59). In contrast to the CSD subtype, in which PD-L2 expression is closely associated with IFN-γ signaling, these subtypes may maintain PD-L2 expression in relatively low-inflammatory contexts. This suggests that non-inflammatory mechanisms may also contribute to PD-L2 regulation (37, 58). DNA methylation at the cg07211259 locus has been associated with PD-L2 transcription. Hypomethylation at this site correlates with elevated PD-L2 mRNA expression across melanoma cohorts (37). However, this association was established mainly through bulk melanoma analyses, and subtype-specific functional validation in acral and mucosal melanoma remains limited.

Although JAK1/2 alterations in acral melanoma may impair canonical IFN-γ–JAK–STAT signaling, preclinical studies suggest that proteasome inhibitors such as bortezomib or MG132 can stabilize IRF1 and induce PD-L2 and HLA-I expression through a JAK-independent mechanism (41).

The therapeutic implications of these mechanisms remain investigational. Epigenetic modulation may potentially increase PD-L2 expression and thereby enhance an immunosuppressive pathway; consequently, epigenetic “unlocking” would require careful assessment of subsequent PD-L2 activity and consideration of combined pathway blockade. Demethylating agents and proteasome inhibitor-based combinations therefore provide a rationale for further preclinical investigation rather than established treatment strategies. In particular, the combination of proteasome inhibition and PD-L2 blockade should be evaluated in acral- and mucosal-specific models before its clinical relevance can be determined.

#### 3.5.3 MITF-low/dedifferentiated phenotype: a potential context for PD-L2 regulation

MITF downregulation is a molecular feature of melanoma cell dedifferentiation (6). Spindle cell morphology is strongly associated with PD-L2 overexpression, occurring in 50% of spindle-cell tumors compared with 5% of tumors with nested morphology (P < 0.0001) (58).

This association may be accompanied by low or absent PD-L1 expression and reduced IFN-γ availability in the tumor microenvironment. However, these observations do not by themselves establish a causal relationship between MITF downregulation, PD-L2 expression, and treatment resistance.

Dedifferentiated melanoma cells may retain altered responsiveness to IFN-γ and exhibit immunosuppressive transcriptional changes, including increased PD-L2 expression in selected models (60). These findings suggest that PD-L2 may contribute to immune suppression in MITF-low tumors, particularly under PD-L1-low or PD-L1-negative conditions. However, whether PD-L2 specifically compensates for PD-L1 blockade through PD-1 and RGMb signaling, and whether this process mediates primary resistance in patients, remains to be established.

Accordingly, targeting PD-L2 or RGMb may represent a potential strategy for further investigation in this phenotype. Its therapeutic value, optimal treatment combination, and biomarker-defined patient population remain investigational.

#### 3.5.4 Host aging and therapy-related stress: potential effects on PD-L2 regulation

Host aging and chemotherapy stress may influence PD-L2 expression and immune resistance through mechanisms that differ from tumor-intrinsic regulation.

Chemotherapy can induce tumor cells to enter a therapy-induced senescence (TIS) state. In preclinical systems, TIS cells have been reported to upregulate PD-L2 and to suppress CD8^+^ T-cell function or recruit myeloid-derived suppressor cells (MDSCs), thereby potentially limiting treatment efficacy and promoting tumor persistence (40). PD-L2 upregulation in the TIS state may partly overlap with the IFN-γ–JAK–STAT pathway but may also depend on transcriptional reprogramming associated with cell-cycle arrest. These findings suggest a therapy-stress-associated regulatory pattern, although its relevance to melanoma immunotherapy requires further validation.

Host aging may also influence melanoma phenotype and treatment response in a sex-dependent manner. In aged male mice, skin fibroblasts with increased reactive oxygen species (ROS) and reduced enhancer of zeste homolog 2 (EZH2) expression secrete higher levels of bone morphogenetic protein 2 (BMP2). BMP2 can induce phenotype switching in melanoma cells, increase AXL expression, and promote a slow-cycling state, which has been associated with resistance to BRAF/MEK-targeted therapy (61, 62).

Although the BMP2-induced AXL^+^ slow-cycling state and the MITF-low dedifferentiated state may partially overlap, they are driven by distinct upstream signals and should not be assumed to represent the same mechanism. The relationship between the BMP2–AXL pathway and PD-L2 regulation remains insufficiently defined.

The BMP2–AXL/MITF-low axis, the IFN-γ–JAK–STAT pathway, and epigenetic regulation may represent distinct regulatory layers connecting tumor state, host factors, and treatment context. PD-L2 blockade, BMP2 inhibition, and age- or sex-informed treatment stratification may provide directions for future translational research. However, these approaches remain investigational, and their efficacy, safety, and appropriate patient selection require prospective validation.

The preceding sections describe the regulatory features of PD-L2 across distinct melanoma subtypes and phenotypic states. For systematic comparison, the proposed upstream drivers, cellular sources, immune microenvironmental contexts, possible PD-L2 functions, therapeutic implications, and current evidence levels are summarized in **Table 2**.

**Table 2:** Regulatory modes and biological functions of PD-L2 across melanoma subtypes/phenotypes.

Subtype/phenotype	Upstream drivers and signals	Major cellular source	Immune microenvironment context	PD‑L2 functional mode	Clinical implication
CSD	IFN-γ–JAK1–STAT1–IRF1 axis; PTPN2 as a negative regulator	Melanoma cells; CD8^+^ TILs	Inflamed or adaptive immune-resistance phenotype	TIL–IFN-γ–PD-L2 axis	Prognostic association in selected cohorts
Acral/Mucosal/MUP	cg07211259 hypomethylation; IFN-γ-independent mechanisms in selected models	Melanoma cells	Variable immune infiltration; PD-L2 evidence remains limited	Possible epigenetic priming of PD-L2 expression	Candidate biomarker requiring prospective validation
MITF‑low/dedifferentiated	MITF loss; enhanced IFN-γ/IRF1 responsiveness	Melanoma cells	Possible immune-excluded or low-inflammation phenotype	Potential compensation in selected PD-L1-low or PD-L1-negative contexts	Candidate therapeutic target requiring validation
Host aging	Stromal ROS–EZH2–BMP2 signaling; possible sex-associated effects	Fibroblasts and melanoma cells	Age-related stromal remodeling and immunosuppression	Possible interaction between BMP2-related switching and PD-L2 regulation	Exploratory age- and sex-associated stratification
TIS	Therapy-induced senescence in selected preclinical models; PD-L2 upregulation in selected models	Melanoma cells	Therapy-stress-associated suppression; possible MDSC involvement	Therapy-stress-associated PD-L2 regulation	Preclinical therapeutic hypothesis
Uveal melanoma	Nuclear PD-L1–EGR1–VEGF signaling; vascular dysregulation	Melanoma cells; PD-L2 source remains uncertain	Vascular and immune exclusion	Possible contribution to residual immune suppression	Evidence currently limited

## 4 PD‑L2 in melanoma immunotherapy: biomarker and therapeutic implications

In adaptive immune resistance, PD-L1 serves as an inducible defense molecule triggered by T-cell attack (63). The dual-ligand resistance model proposed in this Review hypothesizes that PD-L2 may contribute to compensatory immunosuppression in selected contexts where PD-L1 expression or signaling is reduced. We therefore examine whether PD-L2 expression may provide complementary information for immunotherapy response, particularly in selected PD-L1-low or PD-L1-negative contexts. This section synthesizes available clinical and translational evidence relevant to this hypothesis and discusses the potential value of PD-L2 as a biomarker of treatment response or resistance and as a possible therapeutic target in melanoma.

### 4.1 Tissue‑level PD‑L2: epigenetic licensing and subtype‑specific predictive value

PD-L2 expression at the tissue level is influenced by epigenetic regulation, with reported associations with clinical outcomes in cutaneous melanoma. Quantitative methylation-specific PCR (qMSP) and RNA-seq analyses have shown that hypomethylation at the promoter CpG site cg07211259 and higher PD-L2 mRNA expression are associated with longer progression-free survival (PFS) in patients receiving anti-PD-1 therapy (P = 0.030) (37). These observations support the potential relevance of PD-L2-associated biology in melanoma and are consistent with its proposed complementary role in the dual-ligand resistance model. Although current clinical data do not yet establish PD-L2 as an independent predictor of ICB benefit, they provide a rationale for further investigating whether PD-L2 contributes to persistent immune suppression in selected PD-L1-negative melanoma tumors.

PD-L2 expression is not tightly correlated with PD-L1 in some melanoma cohorts, suggesting that PD-L2 may provide information that is complementary to, rather than interchangeable with, PD-L1 status. Transcriptomic analyses have also shown that PD-L2 expression may be more closely linked to Th1/IFNG signatures and CD8^+^ T-cell signaling than PD-L1 in certain tumor types (14, 78). However, high PD-L2 expression may reflect an inflamed or adaptive immune-resistance phenotype rather than directly indicating treatment resistance.

The clinical relevance of PD-L2 has also been explored in retrospective melanoma cohorts. In 147 patients with metastatic melanoma who were evaluated in the absence of therapeutic checkpoint blockade, positive PD-L2 expression was associated with improved overall survival, and PD-L2 alone or in combination with PD-L1 was proposed as a prognostic marker (83). In a cross-cohort analysis, Wang et al. reported that PD-L2 mRNA expression remained associated with overall survival after accounting for TMB and the IFN-γ signature (14). These observations support the potential complementary and prognostic relevance of PD-L2-associated biology in melanoma. However, prognostic association should be distinguished from treatment prediction. These associations do not establish an independent predictive role for PD-L2 in ICB or demonstrate a causal role for PD-L2 in immune suppression. They may instead reflect an inflamed or adaptive immune-resistance phenotype, the abundance and composition of infiltrating immune cells, or other context-dependent features of the tumor microenvironment.

Taube et al. discussed the relationship between PD-L2 expression, CD8^+^ T-cell infiltration, and adaptive immune resistance within the tumor microenvironment (64). In melanoma, this relationship may indicate that PD-L2 is induced in tumors with an ongoing immune response and may provide information complementary to PD-L1 assessment when PD-L1 expression is affected by spatial or temporal heterogeneity. However, increased PD-L2 expression should not automatically be interpreted as evidence of treatment resistance, because it may also reflect an immune-inflamed or adaptive immune-resistance phenotype.

More recently, Sun et al. reported that PD-L2-positive extracellular vesicles (EVs) inhibited CD8^+^ T-cell activation in functional experiments and promoted melanoma growth *in vivo*. In a clinical melanoma study, early dynamic changes in circulating EV-PD-L2 during anti-PD-1-based treatment were associated with treatment response and survival in the discovery cohort, and this association was subsequently examined in an independent cohort receiving anti-PD-1 monotherapy (65). The functional findings provide preclinical support for the potential immunoregulatory activity of EV-PD-L2, whereas the clinical findings remain associative. Together, these data support further investigation of EV-PD-L2 as a dynamic biomarker candidate. Nevertheless, its predictive performance, prespecified and standardized thresholds, reproducibility across treatment regimens, and treatment-specific utility require additional prospective validation, including treatment–biomarker interaction analyses.

## 5 Subtype‑adapted integrative therapeutic strategies targeting PD‑L2 and the clinical translational pathway

Based on the PD-L2 molecular mechanisms, multilayered regulatory networks, and melanoma subtype-specific expression patterns described above, this section proposes a subtype-adapted framework for investigating PD-L2-directed therapeutic strategies in selected melanoma resistance contexts. The aim is to provide a hypothesis-generating basis for future clinical translation, particularly in PD-L1-low or PD-L1-negative refractory melanoma. Most of the proposed combinations are currently supported by mechanistic reasoning, retrospective analyses, or preclinical studies rather than prospective clinical trials. They should therefore be considered investigational strategies within the dual-ligand resistance model, rather than established treatment recommendations.

### 5.1 PTPN2 as a candidate modulator of IFN-γ responsiveness and ICB sensitivity

PTPN2 is an important negative regulator of the JAK–STAT pathway. By dephosphorylating JAK1 and STAT1, increased PTPN2 activity can attenuate IFN-γ signaling, impair antigen-presentation programs, and reduce the immunogenicity of tumor cells. The biological relevance of this pathway is supported by melanoma studies showing that the response to anti-PD-L1 therapy requires JAK1 signaling, whereas JAK2 is dispensable in that experimental context (39). In addition, IFN-γ may exert both antitumor and immunoregulatory effects during melanoma immunotherapy, depending on the cellular and inflammatory context (40). In preclinical melanoma models, genetic or pharmacological inhibition of PTPN2 enhanced IFN-γ–JAK–STAT activity and increased tumor sensitivity to anti-PD-1 immunotherapy, accompanied by improved antigen presentation and antitumor T-cell responses (79, 80).

Within the dual-ligand resistance framework, PTPN2 may therefore represent a broader regulator of the inflammatory state in which PD-L2 expression can be induced. However, current melanoma studies have not directly demonstrated that PTPN2 controls PD-L2 expression or that PTPN2 inhibition creates a specific therapeutic vulnerability to PD-L2/RGMb blockade. Because enhanced IFN-γ responsiveness may also increase the expression of adaptive immune-resistance molecules, including PD-L2, the relationship between PTPN2 inhibition and PD-L2 regulation remains mechanistically plausible but untested.

The available evidence supports PTPN2 inhibition as an investigational immunotherapy-sensitizing strategy in selected melanoma models, rather than as a universal treatment for ICI-resistant patients. Its effects may involve enhanced antigen presentation, chemokine production, and T-cell recruitment, and should not be attributed specifically to PD-L2 modulation without direct experimental evidence. The interaction between PTPN2 activity, PD-L2 expression, and PD-L2/RGMb signaling should therefore be examined in melanoma-specific models before any clinical translation is considered. A dual PTPN2/PTPN1 inhibitor, ABBV-CLS-484, has entered phase I clinical evaluation in patients with advanced solid tumors, both as monotherapy and in combination with a PD-1-targeting agent (90). However, this early clinical development does not establish clinical benefit in melanoma or a PD-L2-specific mechanism of action.

### 5.2 Subtype‑adapted precision therapeutic pathways

The regulatory features and potential contribution of PD-L2 may differ across melanoma subtypes. Multidimensional biomarker stratification may therefore help determine whether PD-L2-directed combinations are applicable to specific melanoma subgroups. The following strategies are proposed as testable therapeutic hypotheses designed to target distinct regulatory contexts within the dual-ligand resistance model: transcriptional modulation in the CSD dedifferentiated subtype, epigenetic modulation in selected acral or mucosal tumors, alternative pathway engagement in JAK1-deficient tumors, and host-microenvironmental intervention in age- and sex-associated settings. The evidence supporting these approaches should be interpreted according to whether it derives from melanoma-specific studies, other tumor types, or preclinical models.

#### 5.2.1 CSD dedifferentiated subtype: transcriptional inhibition and PD‑1/PD‑Ls dual blockade

The CSD dedifferentiated subtype has been associated with high PD-L2 expression, relatively low or heterogeneous PD-L1 expression, and an immune-resistant or less inflamed phenotype in some studies. Its aberrant PD-L2 expression may involve crosstalk between the JAK1–STAT1 and NF-κB pathways (54, 66), while reduced MITF expression contributes to the invasive dedifferentiated phenotype and immune resistance (67). These observations identify the JAK1–STAT1/NF-κB transcriptional axis as a potential regulatory vulnerability in this subtype. However, the extent to which PD-L2 is a causal driver of resistance, rather than one component of a broader dedifferentiation-associated resistance program, remains to be defined.

On this basis, a dual approach combining blockade of both PD-1 ligand pathways with modulation of the JAK1–STAT1 axis could be investigated in biomarker-defined preclinical and early-phase clinical studies. G-quadruplex oligonucleotides, EGCG, and MEK inhibitors represent experimental approaches that may modulate IFN-γ-, IRF1-, or MAPK-related regulation of PD-L2 in selected models (38, 43, 45). Their relative efficacy, specificity, pharmacokinetics, and safety require further evaluation. Bispecific or combined PD-L1/PD-L2 blockade could theoretically inhibit signaling through both PD-1 ligand pathways, but the clinical feasibility and added benefit of this approach remain unestablished. Given the rare 9p24.1 co-amplification context reported in melanoma (32) and the evidence that anti-PD-L1 responses require JAK1 rather than JAK2 signaling in one melanoma model (39), selective JAK2 inhibition combined with PD-L2/RGMb blockade could be explored only as a mechanistic hypothesis in tumors with intact JAK1 function. The efficacy, selectivity, and net immunological effects of this approach remain unknown.

#### 5.2.2 Acral/mucosal subtype: epigenetic unlocking and transcriptional synergistic blockade

Acral and mucosal melanomas may display relatively low inflammatory activity and limited immune infiltration, although their immune phenotype is heterogeneous. In selected tumors, PD-L2 expression may be influenced by promoter methylation at the cg07211259 locus and by inflammatory transcriptional programs (37). However, the specific contribution of PD-L2 to ICI resistance in acral and mucosal melanoma remains insufficiently defined, and general melanoma findings should not be directly extrapolated to all tumors within these subtypes (37, 58, 59).

A biomarker-defined strategy combining epigenetic modulation with PD-L2 pathway blockade could therefore be investigated. In tumors in which promoter methylation limits PD-L2 inducibility, demethylating agents such as decitabine may be evaluated experimentally to modify chromatin accessibility and restore responsiveness to inflammatory signals (37). Because epigenetic reactivation may also increase PD-L2 expression and transiently enhance an immunosuppressive pathway, such intervention should be evaluated together with PD-L2 or PD-1-ligand blockade rather than as a stand-alone treatment. G-quadruplex oligonucleotides or selective JAK-pathway inhibitors could be explored as additional approaches to modulate aberrant PD-L2 transcription in appropriate molecular contexts (38). The direction and clinical relevance of these effects will depend on the baseline methylation state, inflammatory activity, and cellular source of PD-L2. The cg07211259 methylation level may be evaluated as a candidate biomarker, but the previously reported threshold of 9.92% should not be treated as a clinically established positivity cut-off without independent analytical and prospective validation.

#### 5.2.3 JAK1 loss‑of‑function subtype: immunogenicity remodeling and target re‑engagement

The JAK1 loss-of-function subtype is characterized by defects in the IFN-γ–JAK1–STAT1 pathway and may show impaired interferon responsiveness and reduced sensitivity to strategies that depend on intact JAK–STAT signaling (41). These alterations may contribute to a biologically refractory tumorstate, although their clinical prevalence and specific relationship with PD-L2-mediated resistance require further melanoma-specific investigation (44). Chromosome 9p21.3 deletion may also impair immune-related signaling, but its effects on compensatory immunosuppressive pathways remain incompletely understood (68).

The therapeutic challenge in this setting is that pathway defects may reduce tumor immunogenicity while limiting the activity of conventional JAK–STAT-directed interventions. Proteasome inhibitors such as bortezomib have been proposed in preclinical studies to inhibit ubiquitin–proteasome-mediated IRF1 degradation and enhance IRF1 stability through a mechanism that is at least partly independent of JAK kinase activity (41). This approach may partially restore interferon-responsive transcription and potentially re-induce PD-L2 expression, thereby creating a target for subsequent PD-L2 pathway blockade. However, whether this sequence restores meaningful antitumor immunity in JAK1-deficient melanoma remains to be determined.

A combination of immunogenicity remodeling and PD-L2/RGMb blockade could therefore be evaluated experimentally in molecularly defined models. The proposed “remodeling-then-blockade” sequence should be regarded as a testable hypothesis rather than an established treatment order. JAK1 mutation status, residual IFN-response activity, IRF1 protein levels, and PD-L2 induction capacity could serve as exploratory stratification variables in future studies.

#### 5.2.4 Elderly sex‑dimorphic subtype: dual intervention in host microenvironment and immunosuppression

The melanoma microenvironment may display age- and sex-associated differences. In elderly male hosts, dermal fibroblast-derived signals involving reactive oxygen species accumulation and EZH2 downregulation have been reported to increase BMP2 production, which may promote conversion of melanoma cells toward an AXL-positive, slow-cycling or dedifferentiated state and interact with PD-L2-associated immunosuppression (61, 69). These findings provide a rationale for investigating the relationship between BMP2/AXL-mediated phenotypic plasticity and PD-L2 activity in selected models, but they do not yet establish a sex-specific therapeutic algorithm in melanoma.

Accordingly, combined PD-L2 and BMP2/AXL-directed strategies could be explored in appropriate preclinical systems and early-phase clinical studies. The potential value of anti-PD-L2 antibodies in combination with BMP2 inhibition remains investigational, and the efficacy, safety, and optimal treatment sequence have not been established. Whether elderly female patients differ in their response to PD-L2-directed therapy also remains an exploratory question and should not currently be used to select PD-L2 monotherapy or chemotherapy combinations. Fecal microbiota transplantation may influence host–microbiota regulation of the PD-L2–RGMb axis, but this approach remains investigational and requires further evaluation of efficacy, safety, donor-related variability, and mechanism-specific effects (11, 55). Anti-RGMb neutralizing antibodies are likewise at a preclinical stage (70).

Future prospective studies could assess age, sex, BMP2/AXL expression, fibroblast-related signatures, and PD-L2/RGMb activity as exploratory stratification variables. At present, these characteristics should be considered hypothesis-generating factors rather than a basis for treatment selection.

### 5.3 Angio‑immune synergistic sensitization: remodeling the cold tumor microenvironment and compensatory targeting

In uveal melanoma-related studies, nuclear-localized PD-L1 has been proposed to function as a transcriptional regulator of EGR1 and downstream VEGF pathways, thereby contributing to aberrant angiogenesis and a microenvironment characterized by limited immune infiltration and low or heterogeneous PD-L1 expression (33, 71). Conventional anti-PD-L1 antibodies may not fully inhibit nuclear PD-L1 functions, and this possibility provides a rationale for investigating whether PD-L2-mediated suppression becomes more relevant in this context (72). However, the specific contribution of PD-L2 to immune resistance in uveal melanoma remains to be directly established.

A testable therapeutic hypothesis is based on the sequence of microenvironmental remodeling, immune activation, and compensatory checkpoint blockade. First, anti-angiogenic agents such as bevacizumab may remodel abnormal vasculature, improve perfusion, reduce hypoxia, and promote CD8^+^ T-cell infiltration into the tumor parenchyma in selected settings (72, 73). They may also influence PD-L2/PD-1-associated effects on integrin-dependent T-cell adhesion (51). Second, increased immune infiltration and IFN-γ release could alter PD-L2 expression in tumor cells and infiltrating immune cells, with a proportion of PD-L2 potentially being released in extracellular vesicles (65). Third, PD-L2 blockade could then be evaluated for its ability to limit the resulting immunosuppressive signaling.

The proposed “induce-then-block” sequence represents a testable therapeutic hypothesis rather than an established treatment strategy. Vascular and immune remodeling may increase immune infiltration and alter PD-L2 expression, but whether this produces a therapeutically meaningful PD-L2-dependent state remains uncertain. Early clinical studies could evaluate nuclear PD-L1 status, immune-infiltration features, angiogenic signatures, and circulating EV-PD-L2 dynamics as exploratory biomarkers, with initial attention to melanoma contexts showing prominent angiogenic and immune-exclusion features.

### 5.4 Clinical translational pathway and biomarker-guided implementation

In summary, this section presents a hypothesis-driven framework for investigating PD-L2-targeted strategies in selected melanoma tumors with primary resistance to immunotherapy. The proposed strategies are intended to link molecular regulation, melanoma subtype biology, host-microenvironmental factors, and dual-ligand checkpoint activity. They do not imply that PD-L2 is the dominant resistance mechanism in every PD-L1-low or PD-L1-negative tumor, or that the proposed combinations are currently clinically actionable.

The core elements of the subtype-adapted framework, including the proposed mechanisms, experimental therapeutic approaches, candidate biomarkers, current evidence levels, and priorities for preclinical validation and early-phase clinical investigation, are summarized in **Table 3**. This framework may help generate biomarker-driven hypotheses for future studies while placing PD-L2 within the broader landscape of melanoma ICB resistance, including antigen-presentation defects, IFN-signaling abnormalities, T-cell exclusion, immunosuppressive myeloid populations, TGF-β and WNT/β-catenin signaling, metabolic constraints, and tumor heterogeneity.

**Table 3:** Hypothesis-driven experimental approaches for future evaluation of PD-L2-directed strategies in melanoma.

Subtype	Mechanism	Experimental rationale	Combination	Biomarker	Evidence	Priority
CSD	JAK1-STAT1/NF-κB	Transcriptional modulation + PD-1 ligand blockade	G4 oligo + PD-L1/PD-L2 bispecific antibody	MITF-low; p-STAT1	Preclinical	Preclinical validation before any clinical evaluation
Acral/Mucosal	cg07211259 hypomethylation	Epigenetic modulation + transcriptional blockade	Decitabine + JAK2 inhibitor	cg07211259 methylation	Preclinical	Exploratory evaluation after subtype-specific preclinical validation
JAK1 loss-of-function	Impaired IFN-γ response; IRF1 instability	Immunogenicity restoration + checkpoint re-engagement	Bortezomib + anti-PD-L2	JAK1 status; IRF1	Preclinical	Preclinical validation in defined models
Elderly male	BMP2–AXL-associated dedifferentiation	Host–tumor microenvironment modulation	BMP2 inhibitor + anti-PD-L2	Age/sex; BMP2/AXL	Preclinical	Hypothesis-generating stratification
Angio-immune, nPD-L1-positive	Nuclear PD-L1–VEGF signaling	Vascular normalization + PD-L2 blockade	Anti-VEGF + anti-PD-L2	Nuclear PD-L1; EV-PD-L2	Preclinical or early exploratory	Exploratory evaluation in angiogenic or immune-excluded contexts
Selected molecular contexts	PTPN2-mediated JAK–STAT inhibition	Release of negative feedback + checkpoint blockade	PTPN2 inhibition with separate assessment of PD-L2/RGMb activity	PTPN2; JAK-STAT activity	Preclinical	Selected-context sensitization

## 6 Conclusion and future perspectives

PD-L2 emerges as a candidate contributor to immune evasion in melanoma, although its definitive role in primary resistance awaits further experimental confirmation. Current evidence regarding its dual-receptor signaling, potentially reversible inhibitory properties, subtype-specific regulation, and clinical relevance remains fragmented and is derived from heterogeneous experimental and clinical settings. By integrating structural, epigenetic, tumor-subtype, microenvironmental, and clinical evidence, this Review develops a dual-ligand framework for understanding the possible contribution of PD-L2 to immune resistance in melanoma. We use this framework to organize four interconnected areas of uncertainty: molecular mechanisms, biomarker interpretation, subtype heterogeneity, and therapeutic translation. The model is intended to define testable hypotheses and identify priorities for future validation rather than to establish PD-L2 as a universal or dominant resistance mechanism.

As a ligand that can engage both PD-1 and RGMb, PD-L2 has the potential to mediate immunoregulatory effects through two receptor-associated pathways. However, the relative functional contribution and interaction of these pathways in primary melanoma resistance remain uncertain (11). Preclinical mouse studies have suggested an important role for the RGMb axis in specific experimental contexts, but this interpretation is limited by the predominantly murine origin of the evidence, the context dependence of RGMb pathway activity, and the higher affinity of PD-L2 for PD-1, which may allow continued PD-1-associated inhibition even when RGMb is blocked (10, 11, 19, 69). Whether the PD-1 and RGMb pathways act synergistically, redundantly, or hierarchically in human melanoma remains to be determined. This question will require melanoma-specific studies using appropriate humanized, subtype-defined, and genetically controlled models.

The reversibility of PD-L2-mediated immune suppression represents another mechanistic uncertainty. Although studies in non-melanoma systems suggest that PD-L2 may induce reversible T-cell cycle arrest rather than terminal exhaustion (28, 74), functional recovery in the melanoma microenvironment depends on more than checkpoint release alone. T-cell differentiation state, epigenetic programming, and metabolic fitness can influence the extent of recovery (81, 82), and terminally exhausted T cells may retain dysfunctional programs that are relatively resistant to restoration (81). In addition, PD-L2/RGMb-associated regulation may promote local immune tolerance through Treg expansion and IL-10 production in selected models (11, 26, 27, 29). Thus, reversibility should not be regarded as an intrinsic and uniform property of PD-L2 signaling; it is more appropriately viewed as a context-dependent phenotype shaped by inflammatory tone, T-cell state, cellular composition, and metabolic conditions. Whether PD-L2-mediated suppression is more readily reversible than PD-L1-mediated suppression in melanoma remains an open question.

An important upstream question concerns the factors that regulate PD-L2 expression in melanoma. Clinical and transcriptomic studies have associated WNT/β-catenin activity and MITF-low differentiation states with elevated PD-L2 expression (60, 77). However, these associations do not establish a direct causal relationship. Direct β-catenin/TCF binding to the PD-L2 promoter has not been conclusively demonstrated, and the relationship between MITF and PD-L2 may depend on the inflammatory context rather than follow a simple linear regulatory pattern (60). In addition, JAK–STAT-independent signaling, including SOX10–IRF4–IRF1-associated regulation, may indirectly influence PD-L2 transcription (77). Targeted gene-editing, perturbation, and spatially resolved studies will be required to distinguish direct transcriptional control from indirect effects mediated by tumor differentiation or the microenvironment.

Whether PD-L2 can serve as an independent predictive biomarker for immunotherapy remains unresolved. Retrospective studies have associated PD-L2 expression or cg07211259 hypomethylation with favorable outcomes in selected melanoma or immunotherapy-treated cohorts (14, 37), whereas other studies have reported variable findings across patient populations and treatment settings (37, 83). This heterogeneity may reflect differences in PD-L2 antibodies, scoring methods, analytical thresholds, patient ethnicity, melanoma subtype, sample source, treatment regimen, and the timing of tissue collection (64, 83). Importantly, an association with survival or treatment outcome should not be equated with an independent predictive effect or with a causal role in resistance. Prospective, biomarker-stratified studies with standardized assays and treatment–biomarker interaction analyses will be required to determine whether PD-L2 provides predictive information beyond PD-L1, TMB, IFN-γ-related signatures, CD8^+^ T-cell infiltration, and other established features of the tumor microenvironment.

Extracellular-vesicle PD-L2 represents a potentially informative liquid-biopsy marker with distinct temporal features. In the available clinical study, higher baseline EV-PD-L2 levels were associated with poorer outcome, whereas early on-treatment increases in circulating EV-PD-L2 were associated with more favorable treatment response and survival (65). This apparently paradoxical pattern may reflect different biological meanings of baseline abundance and treatment-induced dynamics: baseline EV-PD-L2 may reflect an immunosuppressive tumor burden, whereas an early increase during treatment may represent immune activation, tumor-microenvironment remodeling, or compensatory feedback. These observations should therefore not be interpreted to mean that increased EV-PD-L2 uniformly indicates either resistance or treatment benefit. Standardized detection methods, prespecified thresholds, longitudinal sampling, and validation across treatment regimens and melanoma subtypes remain necessary.

The cg07211259 methylation site is associated with PD-L2 expression in cutaneous melanoma (37), but its clinical applicability remains uncertain. The locus may primarily influence IFN-γ-inducible PD-L2 expression rather than constitutive baseline expression. In addition, bulk sequencing cannot resolve cell-type-specific methylation or distinguish tumor-cell-derived from immune-cell-derived PD-L2. The performance of this marker in acral and mucosal melanoma has also not been adequately validated (37). Although positive PD-L2 expression has been associated with improved overall survival in an independent melanoma cohort (83), it remains unclear whether this association is specifically mediated by cg07211259 hypomethylation or instead reflects a broader inflamed or adaptive immune-resistance phenotype.

Melanoma subtypes may exhibit different patterns of PD-L2 regulation. Cutaneous melanoma may rely more strongly on inflammatory IFN-γ/JAK–STAT signaling to induce PD-L2, whereas some acral and mucosal tumors may show PD-L2 expression in settings with limited inflammatory activity or immune infiltration (58). Bulk cohort analyses have suggested that cg07211259 hypomethylation may contribute to subtype-associated differences in PD-L2 expression (37), but bulk measurements cannot determine the relative contributions of epigenetic priming, inflammatory induction, tumor-cell heterogeneity, and immune-cell composition. Alterations in JAK1/2 or other signaling pathways may further modify these relationships, but the evidence for JAK-independent PD-L2 regulation in individual melanoma subtypes remains incomplete (41). These mechanisms may coexist within the same tumor, and their functional division and clinical significance require subtype-specific, single-cell, and spatially resolved studies.

Host microecology constitutes an additional regulatory dimension. Preclinical mouse models have suggested that the gut microbiota can influence primary anti-PD-1 resistance through mechanisms involving the PD-L2/RGMb axis (11). Clinical studies of fecal microbiota transplantation have also reported restoration or improvement of responses to immune checkpoint blockade in selected melanoma patients (56). However, microbiota-based interventions influence antitumor immunity through multiple parallel mechanisms, including dendritic-cell maturation, cytokine production, antigen presentation, and T-cell recruitment. Therefore, clinical benefit cannot currently be attributed specifically to modulation of the PD-L2/RGMb axis. The physiological role and therapeutic contribution of this pathway in human melanoma remain to be defined.

PD-L2-directed therapy faces an important translational challenge because its potential benefit–risk profile is not yet established. RGMb–PD-L2 signaling contributes to peripheral immune regulation and respiratory tolerance in preclinical models (26). Systemic blockade may therefore theoretically disturb immune tolerance and increase the risk of pulmonary or airway inflammation; however, this risk has not been established in humans. These preclinical observations support careful safety assessment, although animal phenotypes cannot be directly extrapolated to the clinical toxicity of selective pathway blockade. On the efficacy side, combinations of PD-L2 blockade with demethylating agents, proteasome inhibitors, JAK-pathway inhibitors, anti-angiogenic therapy, BMP2/AXL-directed approaches, or microbiota-based interventions remain primarily investigational and are supported mainly by mechanistic or preclinical evidence (37, 39, 41). Their optimal indications, combinations, schedules, and subtype-specific effects remain unknown. Future development should therefore proceed through melanoma-specific preclinical validation, pharmacodynamic biomarker development, and carefully monitored early-phase clinical studies.

The four areas examined in this Review—molecular mechanisms, biomarker interpretation, subtype heterogeneity, and therapeutic translation—indicate that apparent contradictions in the PD-L2 literature may arise from three interacting dimensions: subtype heterogeneity, temporal dynamics, and biological context. For example, PD-L2 expression may reflect either an immune-suppressive mechanism or an inflamed adaptive immune-resistance state, depending on its cellular source, timing, and relationship to other immune variables. By framing these uncertainties as testable hypotheses, the dual-ligand model provides a heuristic framework for integrating fragmented evidence and guiding subsequent validation.

Functional evidence supporting this framework remains largely preclinical, and systematic validation in human melanoma is still insufficient. Key questions include the spatial colocalization of PD-L2 and RGMb, the relative contribution of tumor-cell- and stromal-cell-derived PD-L2, the reversibility of T-cell dysfunction, subtype-specific biomarker performance, the relationship between PD-L2 and other resistance mechanisms, and the safety of pathway-directed interventions. Addressing these questions will require prospective clinical cohorts, single-cell and spatial multi-omics, longitudinal liquid-biopsy studies, genetically controlled melanoma models, and biomarker-stratified early-phase trials.

The Dual-Ligand Resistance Model is therefore best understood as a provisional, hypothesis-driven framework rather than a definitive mechanistic conclusion. Its novelty lies in integrating the distinct PD-L1 and PD-L2 regulatory contexts, the potential PD-1/RGMb dual-receptor biology of PD-L2, and melanoma subtype and host-microenvironmental heterogeneity into a single testable model. The framework does not replace established explanations of melanoma ICB resistance, including antigen-presentation defects, IFN-signaling abnormalities, T-cell exclusion, immunosuppressive myeloid populations, TGF-β and WNT/β-catenin signaling, metabolic constraints, and tumor heterogeneity.

PD-L2 should also be interpreted within the broader network of inhibitory immune checkpoints involved in melanoma immune resistance, including LAG-3, TIM-3, TIGIT, and VISTA (84, 85). These pathways are mediated by inhibitory receptors expressed on T cells and other immune-cell populations and may converge on overlapping programs of T-cell dysfunction, although their cellular sources and molecular mechanisms differ. In melanoma, co-expression of TIGIT and PD-1 has been associated with impaired tumor-antigen-specific CD8^+^ T-cell function (86). TIM-3 upregulation has also been implicated in adaptive resistance to PD-1 blockade, including through STAT3-dependent regulation in melanoma-associated regulatory T cells (87). In addition, increased VISTA expression has been associated with acquired resistance to anti-PD-1 therapy in metastatic melanoma (88). LAG-3 is further distinguished by clinical evidence supporting the benefit of combined LAG-3 and PD-1 blockade in advanced melanoma (89).

Unlike these predominantly inhibitory receptor pathways, PD-L2 is a ligand that combines high-affinity binding to PD-1 with a potential RGMb-associated interaction. This ligand–receptor configuration provides a distinctive conceptual basis for the Dual-Ligand Resistance Model. However, whether PD-L2 contributes independently to melanoma resistance or overlaps functionally with broader T-cell dysfunction programs remains to be determined.

Instead, it proposes that PD-L2 may interact with these mechanisms in selected melanoma contexts and identifies specific questions for future mechanistic and translational investigation.
